# Metabolomic analysis reveals an important role of sphingosine 1-phosphate in the development of HFMD due to EV-A71 infection

**DOI:** 10.1128/aac.01272-24

**Published:** 2024-12-18

**Authors:** Wangquan Ji, Dejian Dang, Guangyuan Zhou, Ling Tao, Tiantian Sun, Dong Li, Cheng Cheng, Huifen Feng, Jinzhao Long, Shuaiyin Chen, Haiyan Yang, Guangcai Duan, Yuefei Jin

**Affiliations:** 1 Department of Epidemiology, College of Public Health, Zhengzhou University 12636 , Zhengzhou, Henan, China; 2 Department of Infection Control, The Fifth Affiliated Hospital of Zhengzhou University 667963 , Zhengzhou, Henan, China; 3 School of Public Health, Xinxiang Medical University 91593 , Xinxiang, Henan, China; 4 Pingyuan Laboratory, Xinxiang, China; IrsiCaixa Institut de Recerca de la Sida, Barcelona, Spain

**Keywords:** hand, foot, and mouth disease, EV-A71, metabolomic analysis, sphingosine 1-phosphate

## Abstract

Hand, foot, and mouth disease (HFMD) is a serious pediatric infectious disease that causes immeasurable physical and mental health burdens. Currently, there is a lack of information on the mechanisms of HFMD severity and early diagnosis. We performed metabolomic profiling of sera from 84 Enterovirus A71 (EV-A71) infections and 45 control individuals. Targeted metabolomics assays were employed to further validate some of the differential metabolic molecules. We identified significant molecular changes in the sera of HFMD patients compared to healthy controls (HCs). A total of 54, 60, 35, and 62 differential metabolites were screened between mild cases and HCs, severe cases and HCs, severe cases and mild cases, and among the three groups, respectively. These differential metabolites implicated dysregulation of the tricarboxylic acid cycle, alanine, aspartate, and glutamate metabolism, and valine, leucine, and isoleucine biosynthesis. The diagnostic panel based on some overlapped differential metabolites could effectively discriminate severe cases from mild cases with an AUC of 0.912 (95% CI: 0.85–0.97) using the logistic regression model. Next, we found the elevation of serum sphingosine 1-phosphate (S1P) level in EV-A71 infection mice, which was similar to clinical observation. Importantly, after blocking the release of S1P by MK571, the clinical symptoms and survival of mice were significantly improved, involving the reduction of leukocyte infiltration in infected brain tissues. Collectively, our data provided a landscape view of metabolic alterations in EV-A71 infected children and revealed regulating S1P metabolism was an exploitable therapeutic target against EV-A71 infection.

## INTRODUCTION

Hand, foot, and mouth disease (HFMD) is a common pediatric illness characterized by red spots on the hands and feet, as well as painful spots in the mouth and throat (1). While most HFMD cases are mild and self-limiting, a small proportion of severe cases can eventually progress to cardiorespiratory failure and even death (2). Human enterovirus A71 (EV-A71), a major pathological cause of severe HFMD, was first isolated from the stool of a child with encephalitis in 1969 (3). EV-A71 has also been associated with several outbreaks of neurological complications across the Asia-Pacific region (4, 5). To date, the mechanism behind the severe complications caused by EV-A71 remains unclear. Although an inactivated EV-A71 vaccine was introduced in 2015 (6), severe HFMD is still an important public health issue globally due to the high incidence of HFMD. Therefore, there is an urgent need to identify more effective and convenient biomarkers for diagnosing severe HFMD and to develop strategies for its control and treatment.

In recent years, the development of systems biology technologies, such as metabolomics, has provided more effective tools for studying disease pathogenesis and identifying new diagnostic indicators. Metabolism plays a crucial role throughout the entire life process, and the occurrence and development of any disease can disrupt the normal bodily metabolism, leading to changes in metabolites (7). Comparing and analyzing these metabolic changes can enhance our understanding of disease mechanisms, assisting clinical diagnosis and drug development. EV-A71 has been demonstrated to reprogram glucose metabolism through the activation of glycolytic pathways, and inhibition of glycolysis effectively attenuates the viral infection (8). In EV-A71-infected neural progenitor cells, perturbations in key metabolic pathways, including the pentose phosphate pathway, gluconeogenesis, and glutamate metabolism, were identified as the most significantly affected (9). Amino acid metabolism plays an essential role in the modulation of both innate and adaptive immune responses. In Vero cells infected with EV-A71, several amino acids, notably glutamate and aspartate, exhibited pronounced changes in response to varying viral doses. Other critical metabolic pathways, including glycolysis and the tricarboxylic acid cycle (TCA), were also significantly modulated (10). In an EV-A71-infected murine model, disruption of arginine/ornithine metabolism emerged as a key determinant in the pathogenesis of cytokine storms, which are characteristic of severe HFMD cases (11).

Sphingolipids are essential and ubiquitous components of all eukaryotic membranes, and they can be metabolized into signaling molecules. Sphingosine 1-phosphate (S1P) derived from membrane sphingolipids can evoke multiple cellular processes through any of the five G protein-coupled receptors (S1P receptor 1–5, S1PR1-5) or through intracellular targets, including cell growth, migration, apoptosis, and immune cell functions (12). Notably, the S1P pathway plays critical roles in immune response, serving as a dynamic regulator of viral infection (13). For example, in influenza virus infection, plasma S1P levels increased in infected mice, which was associated with elevated gene expression of sphingosine kinase-1 (14). An S1PR agonist can provide protection against pathogenic influenza viruses by suppressing cytokine storms (15). Additionally, in coxsackievirus B3-induced myocarditis, FTY720, a structural analog of S1P, can alleviate symptoms and inhibit viral replication by regulating S1P receptors and AKT/caspase-3 pathways (16). However, the roles of S1P in EV-A71-induced HFMD remain largely unknown.

In the present study, we utilized liquid chromatography-mass spectrometry to analyze metabolites in the plasma of healthy children, as well as in those of mild and severe HFMD cases infected with EV-A71. A total of 54, 60, 35, and 62 differential metabolites were identified when comparing mild cases to healthy controls (HCs), severe cases to HCs, severe cases to mild cases, and across all three groups, respectively. Alterations in the TCA cycle, as well as in the biosynthesis of valine, leucine, and isoleucine, and the metabolism of alanine, aspartate, and glutamate, are implicated in the progression of disease severity in HFMD. Our findings elucidated the metabolic mechanisms were involved in the progression of HFMD severity and identified as potential diagnostic biomarkers. At the same time, we evaluated the efficacy of regulating S1P by targeting S1P metabolism and S1PR1 signaling pathway, which may pave the way for new therapeutic options for HFMD.

## RESULTS

### Altered plasma metabolic profiles of EV-A71 patients

**Fig 1:**
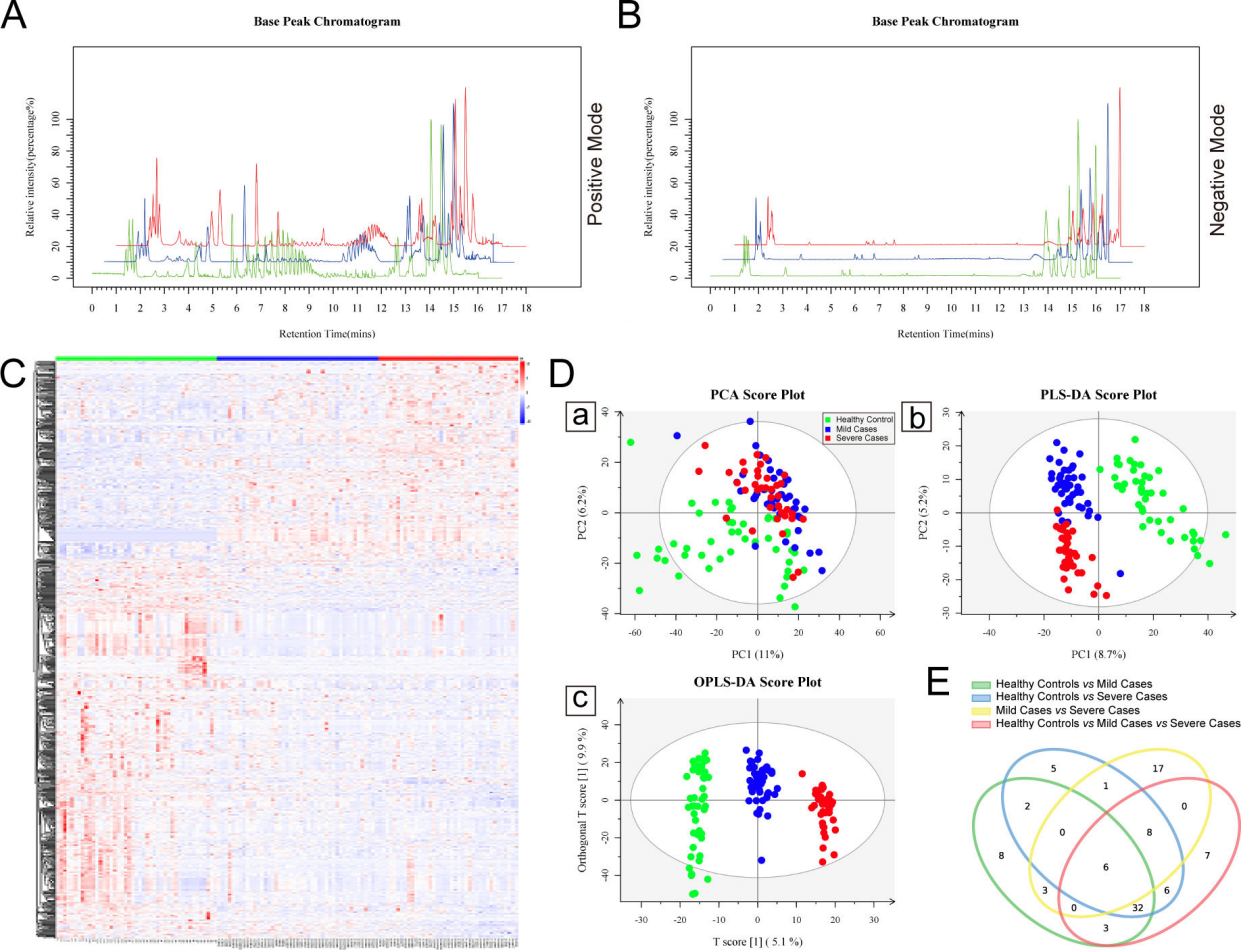
Metabolomic profiling of HFMD plasma. (**A and B**) The positive and negative ion mode chromatograms of the three groups of samples. (**C**) The cluster analysis of metabolic profiles by heatmap among different groups. Red is increasing, blue is decreasing across each row. (**D-a**) PCA score plots, (**D-b**) PLS-DA score plots, and (**D-c**) OPLS-DA score plots based on the data from the HCs (green), mild cases (blue), and severe cases (red). (**E**) Venn diagram analysis identifies differentiated metabolites across four comparison strategies (HCs vs mild cases; HCs vs severe cases; mild cases vs severe cases; HCs vs mild cases vs severe cases).

The characteristics and laboratory indicators of the participants are shown in Table S1 of Supplementary file S1. Among the 129 participants, the HCs, mild cases, and severe cases were comparable in gender and age. Compared to mild cases, severe cases showed significant changes in the number of white blood cell (WBC), neutrophils, and the levels of total proteins, hypersensitive C-reactive protein (hs-CRP), glucose, and globulin. To directly observe the differences in metabolic profiles between EV-A71 patients and HCs, we compared the ultrahigh performance liquid chromatography-mass spectrometry base peak intensity chromatograms of HCs, mild patients, and severe patients. In both positive and negative ion mode chromatograms of the three groups of samples, the chromatographic peaks were all well resolved (Fig. 1A and B). In the 6–10 min and 10–12 min sections of the positive ion chromatograms, as well as the full spectrum of the negative ion chromatograms, the profiles of HCs differed significantly from those of mild and severe patients. Additionally, differences were observed between the mild and severe groups. Further cluster analysis using a heatmap (Fig. 1C) showed that the metabolic profiles of the mild and severe patients were significantly changed compared to HCs, and there were also distinct differences between the mild and severe groups. These metabolomics profiles were able to distinguish EV-A71 infection from HCs, and can also differentiate severe patients from HCs and mild patients, by using PCA (Fig. 1D-a), PLS-DA (Fig. 1D-b), and OPLS-DA model (Fig. 1D-c). Taken together, our results suggest that EV-A71 infection leads to alterations in plasma metabolic profiles among HCs, mild and severe EV-A71 patients.

### Differential metabolic profiles and metabolic pathways across HCs, mild and severe EV-A71 patients

**Fig 2:**
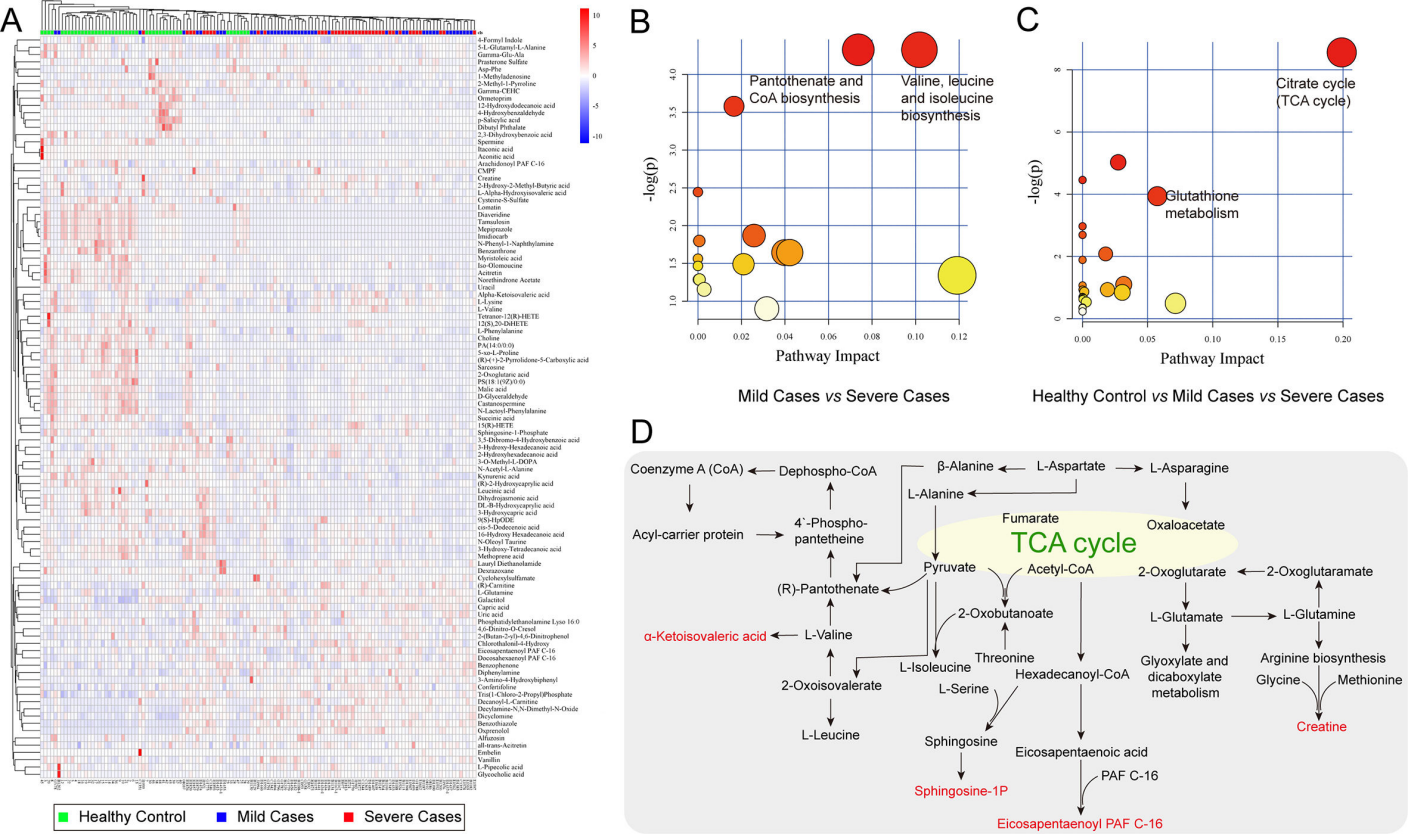
Differential metabolic profiles and metabolic pathways across HCs, mild and severe EV-A71 patients. (**A**) Heatmap visualization of hierarchical clustering based on significantly altered metabolites (VIP > 1 and *P* < 0.05 and fold changes ≥ 1.5 or ≤0.667 between groups). Metabolic pathway analysis was launched using identified differential metabolites. The color and size of each circle are based on its *P* value and pathway impact value, respectively. (**B**) Mild cases vs severe cases; (**C**) HCs vs mild cases vs severe cases. (**D**) Metabolic pathway map of the major differential metabolites.

After filtering the metabolites based on the criteria, hierarchical clustering revealed distinct metabolic signatures among groups. Finally, a total of 54, 60, 35, and 62 differential metabolites were identified between mild cases and HCs (Fig. S1A), severe cases and HCs (Fig. S1B), severe cases and mild cases (Fig. S1C), and among the three groups (Fig. 2A), respectively. Notably, six differential metabolites overlapped across all four comparison strategies (Fig. 1E). Metabolic pathway analysis of significantly altered metabolites was performed with KEGG enrichment (Fig. S1D and E). This analysis revealed that changes in the TCA cycle, valine, leucine, and isoleucine biosynthesis, alanine, aspartate, and glutamate metabolism, were the most pronounced in EV-A71 infection (Fig. 2B and C). We illustrated the transformation relationships of some differential metabolites (Fig. 2D). Apparently, multiple metabolic pathways were involved in the development of HFMD severity, consistent with the above findings.

### Differential metabolites as possible indicators of EV-A71-related severe outcome

**Fig 3:**
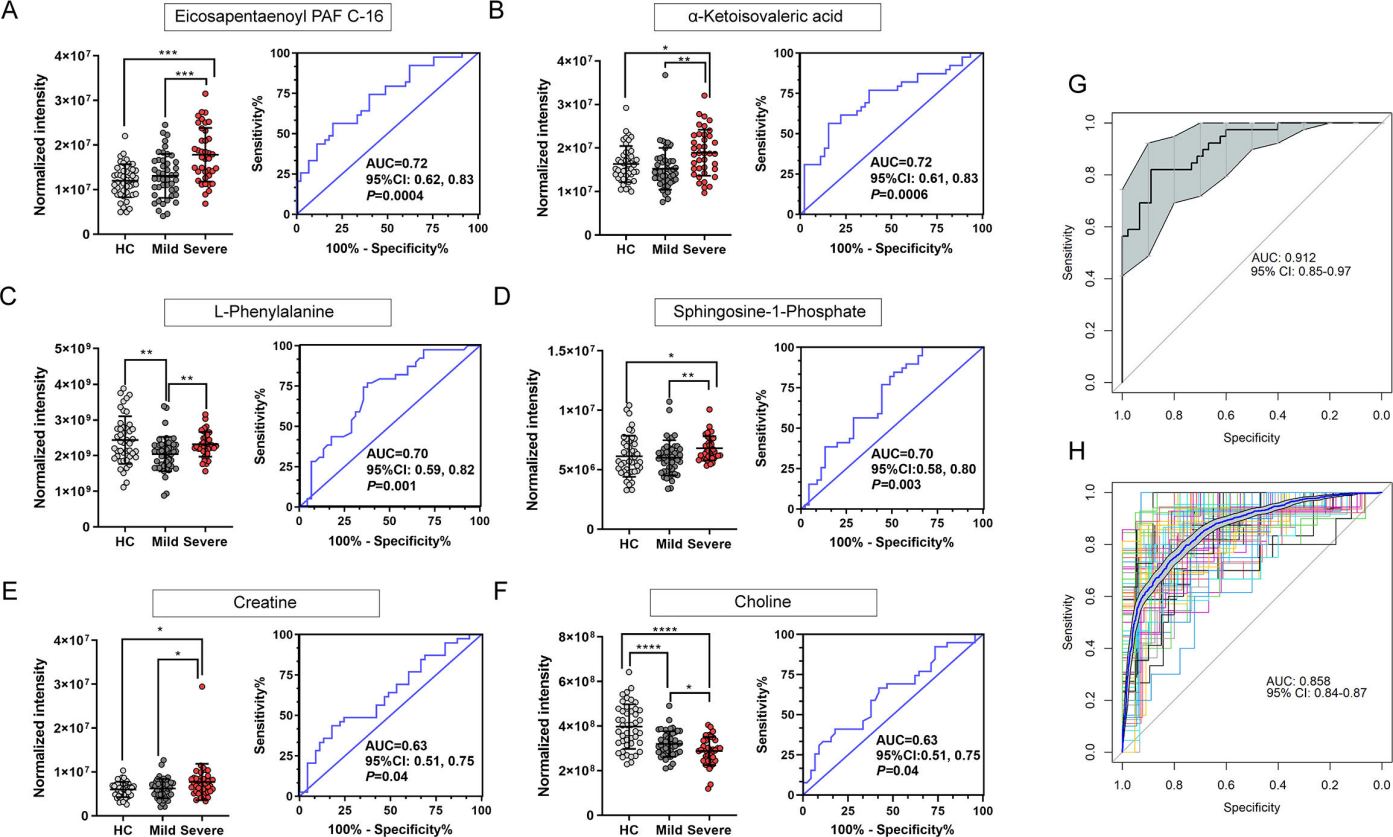
Potential predictive values of differential metabolites for the onset of severe EV-A71 infection. Six elected metabolites that significantly differed between mild, severe, and HCs. (**A**) Eicosapentaenoyl PAF C-16, (**B**) α-ketoisovaleric acid, (**C**) L-phenylalanine, (**D**) S1P, (**E**) creatine, and (**F**) choline. The logistic regression model was constructed for calculating mild group and severe group diagnosis score. (**G**) The corresponding ROC of the logistic regression model. (**H**) The holdout cross-validation analysis for 100 times. ROC curves showing the performance of models. AUC indicates the area under the curve, and CI indicates the confidence interval. *P < 0.05; **P < 0.01; ***P < 0.001; ****P < 0.0001.

Based on the metabolites with significant changes, ROC analysis was conducted to evaluate their sensitivity and specificity in discriminating severe from mild EV-A71 patients. Six metabolites with potential diagnosis value for EV-A71-related HFMD severity (AUC > 0.6) were identified: eicosapentaenoyl platelet-activating factor C-16 (PAF C-16) (Fig. 3A), α-ketoisovaleric acid (Fig. 3B), L-phenylalanine (Fig. 3C), S1P (Fig. 3D), creatine (Fig. 3E), and choline (Fig. 3F). These metabolites are involved in neuromodulation (L-phenylalanine, choline, S1P, creatine), and the biosynthesis of valine, leucine, and isoleucine (α-ketoisovaleric acid). We speculate that these metabolites may play an important role in the development of severe HFMD. We then investigated whether these differential metabolites could be used to predict clinical development. We applied several classical models to calculate diagnosis score for mild and severe groups. The diagnostic performances of the logistic regression model (Fig. 3G and H), Bayesian model (Fig. S2A), Random forest model (Fig. S2B), and Artificial neural networks analysis (Fig. S2C) were evaluated and compared. Optimally, the corresponding AUC of the logistic regression model was 0.912 (Fig. 3G), and the holdout cross-validation analysis showed the AUC based on logistic regression was 0.858 (95% CI: 0.84–0.87) (Fig. 3H). These results indicate that these differential metabolites can be used to predict clinical development. The diagnostic panel based on the logistic regression model could more effectively discriminate severe cases from mild cases.

### Quantification of unique plasma metabolites in the development of disease severity caused by EV-A71

**Fig 4:**
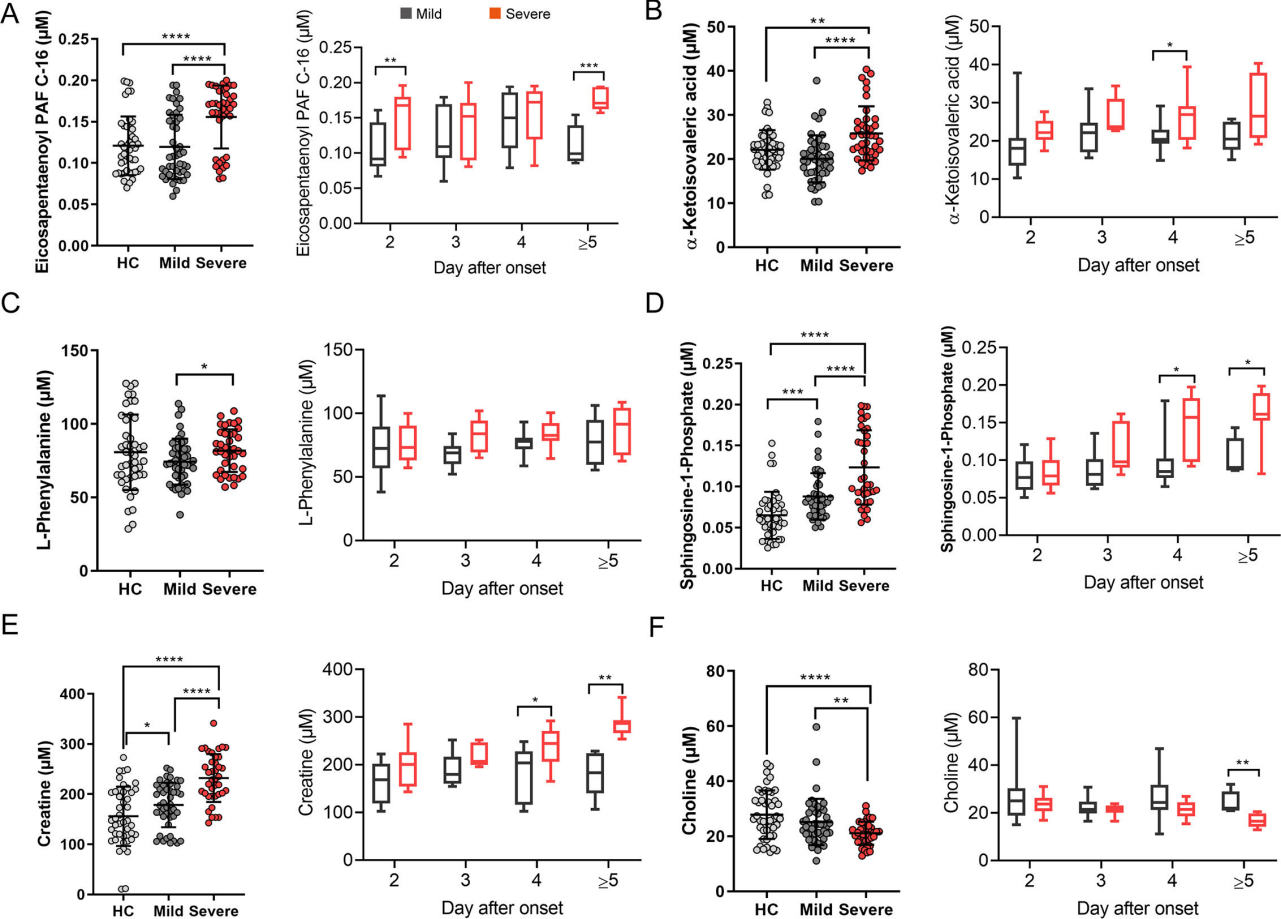
Quantification of unique plasma metabolites of severe EV-A71 patients. The quantification of unique plasma metabolites in different groups (mild, severe, and HCs) and subgroups between mild and severe cases. Mild and severe patients were then divided into subgroups according to the number of days after onset: 2, 3, 4, and ≥5 days. The number of mild cases in each subgroup was 16 (2 days after onset), 13 (3 days), 11 (4 days), and 5 (≥5 days). The number of severe cases in each subgroup was 12 (2 days), 7 (3 days), 12 (4 days), and 8 (≥5 days). (**A**) Eicosapentaenoyl PAF C-16, (**B**) α-ketoisovaleric acid, (**C**) L-phenylalanine, (**D**) S1P, (**E**) creatine, and (**F**) choline. *P < 0.05; **P < 0.01; ***P < 0.001; ****P < 0.0001.

To further evaluate the role of these overlapped six differential metabolites in the development of disease severity, we quantified their concentrations using UHPLC-TQ-MS/MS. The change trends of these metabolites were consistent with the results of non-targeted metabolomics. Compared to mild EV-A71 patients, the concentrations of eicosapentaenoyl PAF C-16 (Fig. 4A), α-ketoisovaleric acid (Fig. 4B), L-phenylalanine (Fig. 4C), S1P (Fig. 4D), and creatine (Fig. 4E) were significantly increased, while the concentration of choline (Fig. 4F) was significantly reduced. To study the differential metabolites in EV-A71 pathogenesis, EV-A71 patients were divided into different subgroups based on the days after onset. As shown in Fig. 4A, the concentration of eicosapentaenoyl PAF C-16 of severe patients was significantly elevated at 2, ≥ 5 days after onset compared to mild patients. As shown in Fig. 4B, the concentration of α-ketoisovaleric acid of severe patients was significantly elevated at 4 days after onset compared to mild patients. Additionally, the concentrations of S1P and creatine of severe patients were significantly increased at 4, 5 days after onset compared with mild patients. These metabolites with dynamic changes may be associated with the development of disease severity.

### The correlation of S1P and clinical indicators

**Fig 5:**
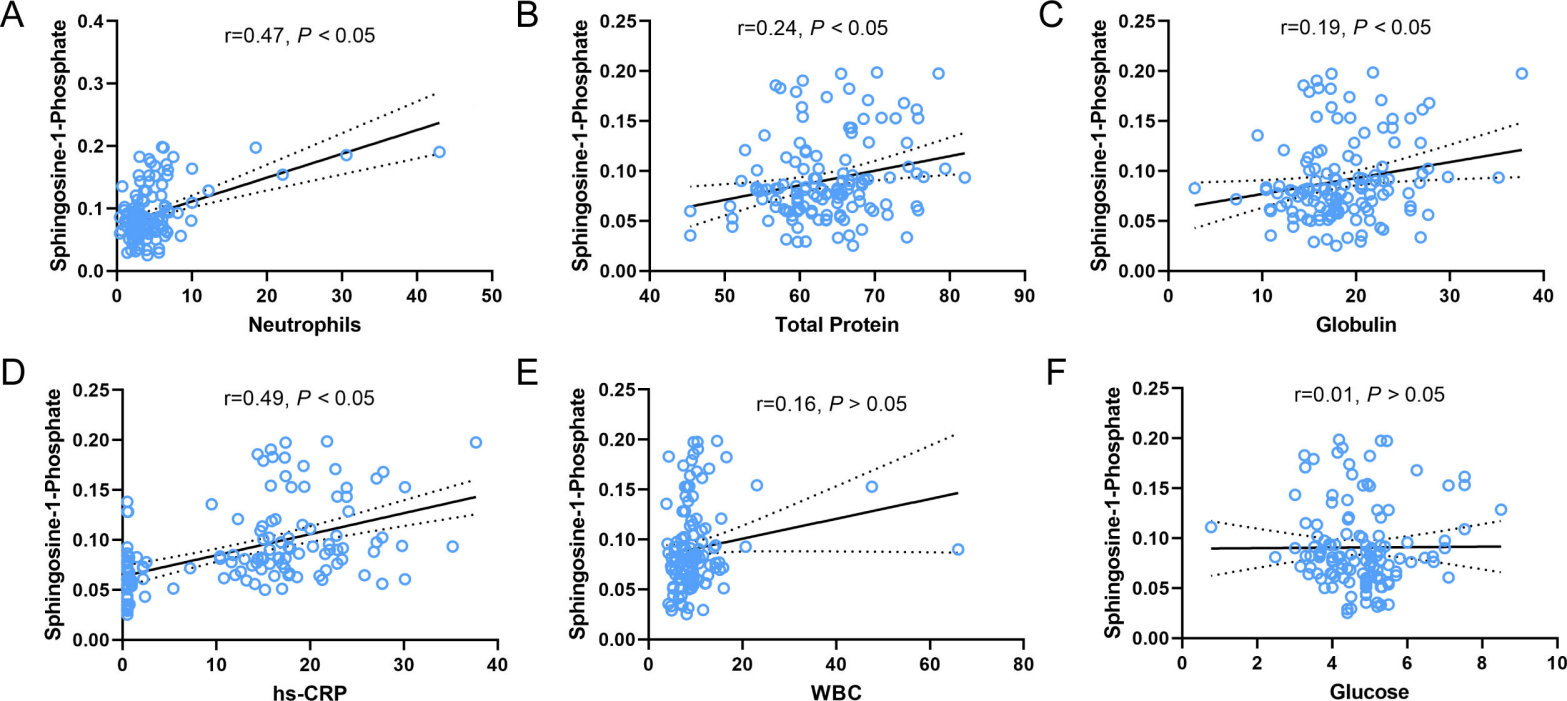
Correlation analysis between S1P and some clinical indicators. The concentration of serum S1P showed positive correlation with (**A**) neutrophils, (**B**) total protein, (**C**) globulin, (**D**) hs-CRP, (**E**) WBC, and (**F**) glucose. The *r* value represents the correlation coefficient between the two variables.

Considering that the central nervous system is the main target of HFMD and that the brain contains the highest concentration of sphingolipids in the body (17, 18), S1P may emerge as a potential target. To further explore the correlation between S1P and clinical progression, Pearson correlation analysis was performed between S1P and several important clinical indicators. S1P concentration showed a positive correlation with neutrophils (Fig. 5A), total protein (Fig. 5B), globulin (Fig. 5C), and hs-CRP (Fig. 5D), with statistically significant. It should be emphasized that the correlation coefficient between S1P concentration and the number of neutrophils and hs-CRP was almost 0.5, indicating that S1P was closely related to inflammation response. Although S1P was also positively correlated with WBC (Fig. 5E) and glucose (Fig. 5F), there was no statistical difference. Additionally, we also found correlations between other differential metabolites and clinical indicators. A positive correlation was observed between creatine concentration and WBC (Fig. S3A), hs-CRP (Fig. S3B), and neutrophils (Fig. S3C). Eicosapentaenoyl PAF C-16 concentration was positively correlated with hs-CRP (Fig. S3D), glucose (Fig. S3E), as well as total protein (Fig. S3F). Conversely, an inverse correlation was simultaneously shown between choline concentration and neutrophils (Fig. S3G), hs-CRP (Fig. S3H), as well as total protein (Fig. S3I). Taken together, these results indicate that the differential metabolites are correlated with various clinical indicators. Particularly, S1P may be associated with the development of disease severity.

### Regulating S1P metabolism alleviates EV-A71-induced disease severity in mice

**Fig 6:**
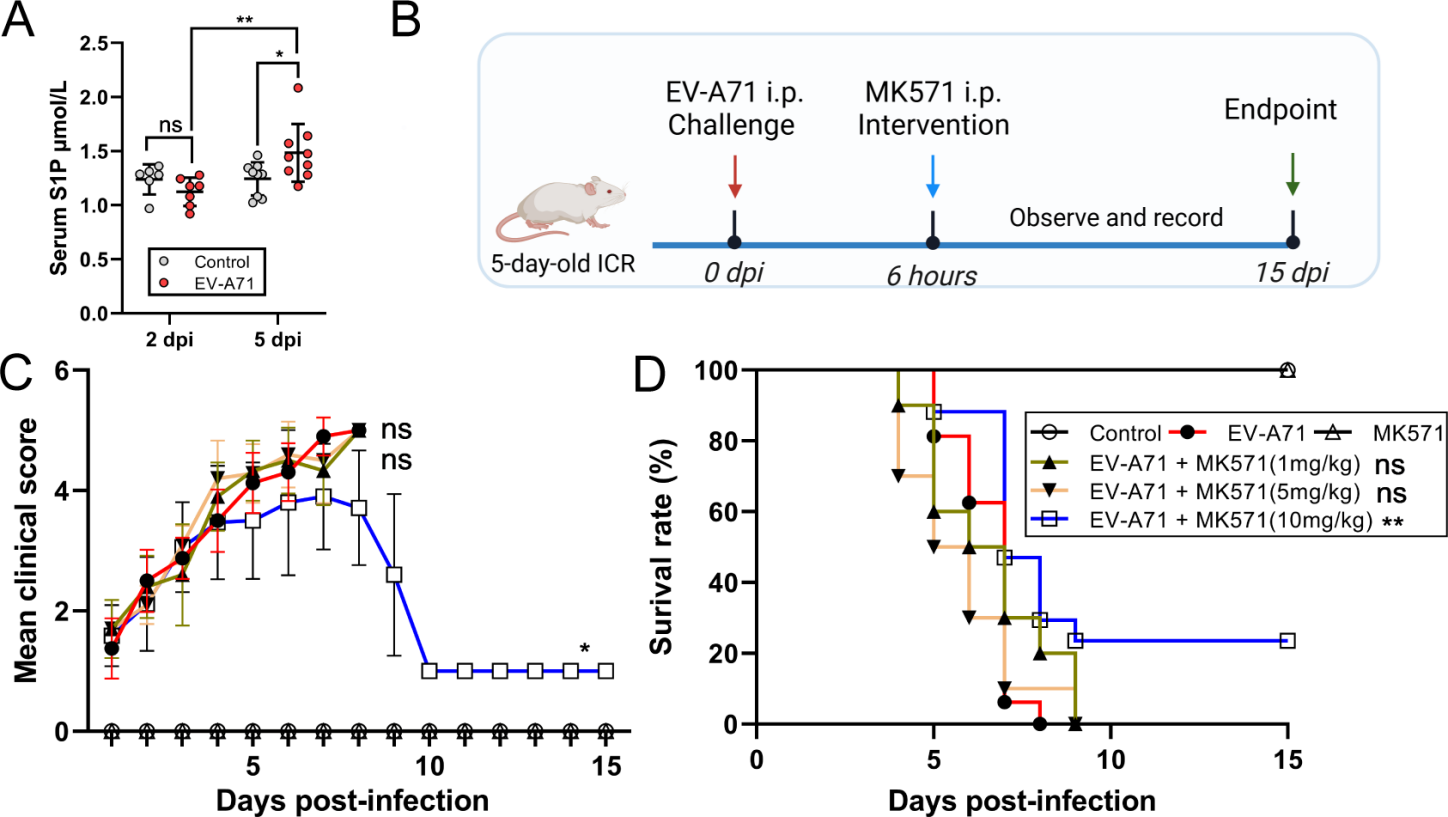
Regulation of S1P metabolism can improve the survival rate of infected mice. (**A**) Serum S1P levels of infected and control mice were measured by ELISA at 2 and 5 dpi (*N* = 6–9 per group). (**B**) A schematic diagram to explain the animal experiment. Following treatment as indicated in the schematic diagram, (**C**) mean clinical score and (**D**) survival rate were monitored daily for 15 days (*N* = 10–17 per group, vs EV-A71). Clinical scores were defined as follows: 0, healthy; 1, reduced mobility; 2, ruffled hair, hunchbacked, or ataxia; 3, weight loss; 4, limb weakness; and 5, dying or death. *P < 0.05; **P < 0.01; ns, no statistical differences. Created with BioRender.com.

Metabolites can act as signaling molecules to regulate important pathophysiological processes. We first measured the serum S1P levels based on an EV-A71 infection mouse model and observed the elevation at 2 and 5 dpi (Fig. 6A), which was consistent with clinical observation (Fig. 4D). We further evaluated the alterations in other metabolites in the infected mice. The analysis revealed that serum concentrations of L-phenylalanine and choline in the infected mice remained largely unchanged, potentially highlighting species-specific differences in metabolic responses to infection between humans and animals (Fig. S4A and C). Notably, consistent with findings from human participants, serum creatine levels in the infected mice were significantly elevated following infection (Fig. S4B). Next, we evaluated the therapeutic effect of MK571, an inhibitor of S1P, following the established protocol (Fig. 6B). MK571 could inhibit both constitutive and antigen-stimulated S1P release. MK571 exhibits a wide range of safe dosage levels. Notably, significant cytotoxic effects are observed only at concentrations exceeding 25 µM (Fig. S4D). Low-dose interventions had minimal effects. But at a dose of 10 mg/kg, the mean clinical score of infected mice was reduced, and the survival rate increased from 0% to 23.5% (Fig. 6C and D). Therefore, targeting S1P metabolism appears to be an effective intervention for managing EV-A71 infection.

### Targeting S1P-metabolism may alleviate infiltration of leukocyte

**Fig 7:**
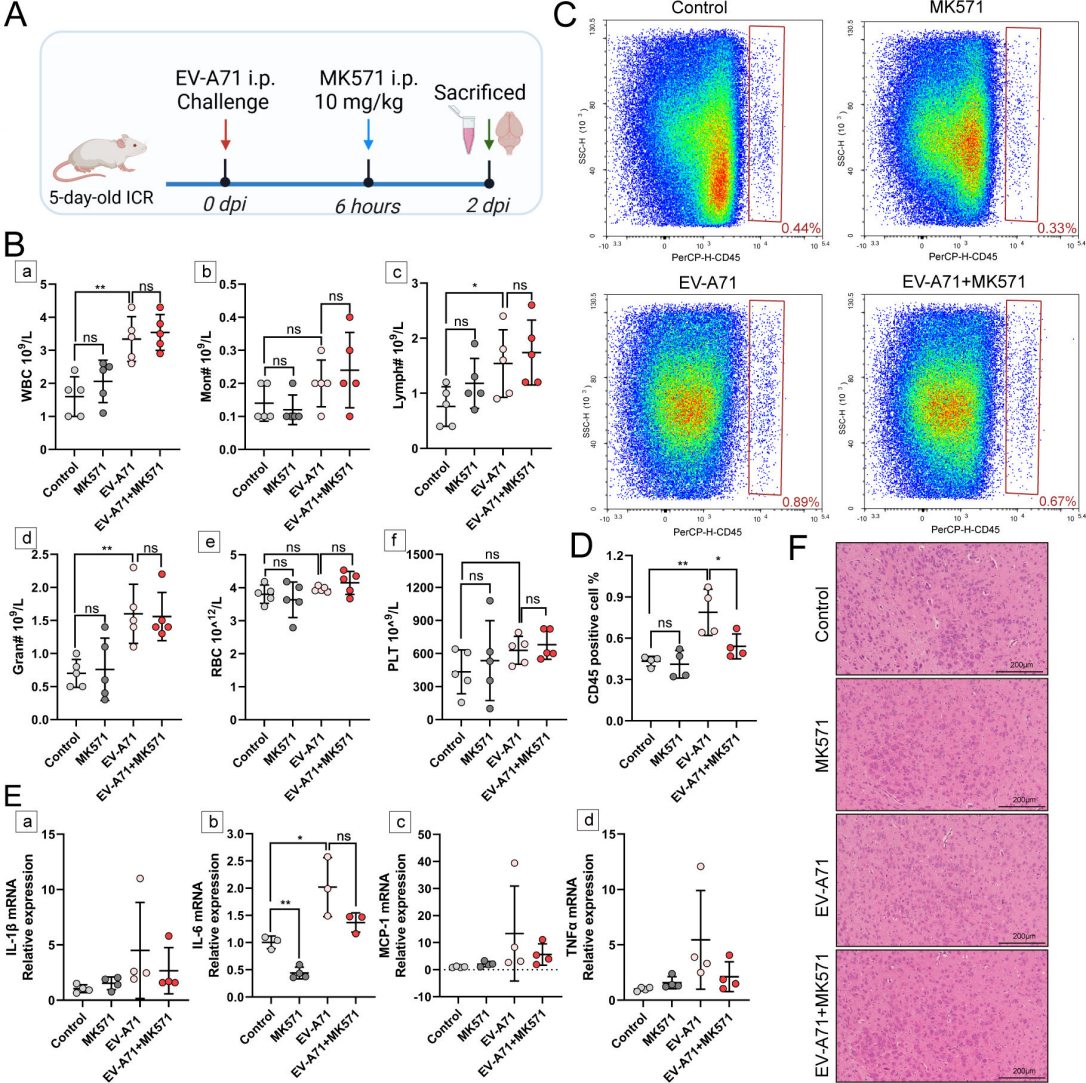
Targeting S1P-metabolism may regulate immune function in the brain. (**A**) A schematic diagram to indicate the procedures for intervention and collecting samples. (**B**) The blood routine was detected by an automatic blood cell analyzer at 2 dpi (*N* = 5 per group). The main detection indicators included a: WBC, b: monocyte (MON), c: lymphocyte (LYM), d: granulocyte (Gran), e: red blood cell (RBC), and f: platelet (PLT). (**C**) The numbers of CD45 positive cells in brain tissues were measured by flow cytometer at 2 dpi (*N* = 4 per group). (**D**) The results of flow cytometry were analyzed. (**E**) qPCR was conducted to measure the gene expression levels of some typical inflammatory cytokines (a, IL-1β; b, IL-6; c, MCP-1; d, TNFα) in the brains of EV-A71-infected and mock mice with MK571 interventions at 2 dpi (*N* = 3–4 per group). (**F**) Pathological changes in the brains of mice at 2 dpi were evaluated by H&E staining. The scales are indicated in these images. *P < 0.05; **P < 0.01; ns, no statistical differences. Created with BioRender.com.

Given that the mice exhibited significant clinical symptoms in the later stages of infection, we focused on understanding how MK571 works in the early stage (Fig. 7A). Infected mice displayed significantly reduced serum S1P levels following MK571 treatment (Fig. S4E). This observed therapeutic benefit is attributed, at least in part, to the partial suppression of serum S1P levels. Blood routine analysis revealed that the drug did not directly affect the number of different types of peripheral immune cells, nor did it affect the counts of RBC and PLT (Fig. 7B). Flow cytometry demonstrated that MK571 reduced leukocyte infiltration in brain tissue (Fig. 7C and D), which was accompanied by a decrease in the transcriptional levels of several key cytokines although these changes did not reach statistical significance (Fig. 7E). Furthermore, histopathological examination indicated an absence of significant inflammatory infiltrates or tissue abnormalities in the brain tissues across all four groups of mice in the early stage (Fig. 7F). Because of the low viral load in brain tissues in the early stage, we could not detect the effect of drugs on antiviral capacity. At 5 dpi, no statistically significant differences in viral loads were observed between the EV-A71 infection group and the MK571 intervention group (Fig. S4F), indicating that MK571 did not directly reduce viral replication. While MK571 treatment was associated with a more pronounced decline in cytokine expression (Fig. S5A through D) and some improvement in brain pathological damage, it was unable to completely prevent the pathological changes in brain tissue (Fig. S5E). Meanwhile, considering that S1PR1 is closely associated with immunity, we further investigated the role of S1PR1 in HFMD progression using a highly selective S1PR1 agonist, SEW2871 (Fig. S6A). Unfortunately, although SEW2871 partially decreased the clinical score of infected mice, it did not improve survival rates (Fig. S6B). The drug could reduce the transcriptional levels of some cytokines in brain tissue but did not prevent the increase in cytokines following viral infection (Fig. S6C). Thus, targeting S1P-metabolism rather than S1PR1 signaling might ameliorate EV-A71-associated encephalitis.

## DISCUSSION

Prophylactic and therapeutic agents against HFMD remain inadequately established. Early identification of severe HFMD in children and timely intervention can significantly reduce mortality (19). Metabolomics, due to its sensitivity, can detect subtle biological pathway alterations, offering insights into mechanistic studies and disease diagnosis (20). Furthermore, metabolic dysregulation plays a crucial role in the development and progression of HFMD (21). In the present study, we explored the potential application of differential metabolites in predicting and diagnosing severe HFMD early. Additionally, we assessed the effectiveness of targeting S1P metabolism and S1PR1 signaling pathways, highlighting the pivotal role of S1P in HFMD development.

A significant impact on the serum metabolome, influencing several metabolic pathways to varying degrees, has been observed in HFMD patients (22). In this study, we identified 54, 60, 35, and 62 differential metabolites across four comparison strategies, respectively. These alterations highlight the broad metabolic implications of HFMD, reflecting its complex effects on biochemical processes essential for both viral pathogenesis and host responses. Specifically, we observed prominent changes in the TCA cycle, as well as in alanine, aspartate, and glutamate metabolism in response to EV-A71 infection. The TCA cycle plays a central role in viral infection, replication, viral pathogenesis, and anti-viral immune responses (23). The TCA cycle plays a central role in the complete oxidation of acetyl-CoA, generating intermediates essential for ATP production and supporting various anabolic pathways, including amino acid synthesis. These TCA-derived intermediates regulate a broad range of critical biological processes. Both biosynthesis and energy production within the TCA cycle are indispensable for viral infections. Key metabolites, such as citrate, succinate, fumarate, and itaconate (which is synthesized from the TCA cycle intermediate cis-aconitate), are integral to maintaining pro-inflammatory/anti-inflammatory balance. These metabolites contribute to both antiviral immune responses and the inflammation associated with virus-induced pathologies (23). The pathway of alanine, aspartate, and glutamate metabolism is critical for neurotransmission (24). Furthermore, we discovered that the biosynthesis of branched chain amino acids (BCAAs, including valine, leucine, and isoleucine) was a key pathway involved in the development of HFMD severity. BCAAs are integral to various physiological functions such as the regulation of neurotransmitter synthesis and immunity (25). The accumulation or insufficiency of BCAAs can contribute to neurologic dysfunction and irreversible brain damage (26). Previous studies have also noted elevated serum levels of myo-inositol, valine, leucine, lysine, isoleucine, and 3-hydroxybutyrate in children with HFMD (22). Based on the ROC analysis attained from this study, the identified differential metabolites could serve as valuable diagnostic markers for severe HFMD. The diagnostic panel utilizing a logistic regression model was particularly effective, distinguishing severe cases from mild cases with an AUC of 0.91. Future prospective studies and further validation can help refine and enhance the accuracy of this model.

We found that eicosapentaenoyl PAF C-16, α-ketoisovaleric acid, L-phenylalanine, S1P, and creatine were enriched, while the concentration of choline was depleted in severe HFMD patients. Eicosapentaenoyl PAF C-16 can be formed by incorporation of eicosapentaenoic acid into lyso-PAF C-16, which can serve as a substrate for PAF C-16 formation via the remodeling pathway. During infection, PAF C-16 is released by various cells, including neutrophils, endothelial cells, mast cells, and monocytes upon stimulation (27). PAF C-16 can trigger downstream signaling pathways that involve phospholipases, kinases, and the production of multiple cytokines (28). Valine is converted into α-ketoisovaleric acid by BCAA aminotransferase in cytoplasm or mitochondria. Finally, these intermediates are catabolized to succinyl-CoA, which is used in the TCA cycle for ATP production and gluconeogenesis (29). The valine/α-ketoisovaleric acid catabolic pathway also plays a pivotal role in regulating platelet activation (30). Besides, the competition between BCAAs and aromatic amino acids (like phenylalanine) may affect the synthesis of neurotransmitters, particularly dopamine, norepinephrine, and serotonin. Correspondingly, plasma concentrations of catecholamines are significantly elevated in severe HFMD patients compared to mild and healthy individuals (31, 32). The reactions involving the amino acids arginine, glycine, and methionine could produce creatine, which is important for resynthesizing ATP, particularly during times of increased metabolic demand (33). Creatine’s mechanisms of action include rapid energy provision and the reduction of reactive oxygen species formation. Therefore, the increase in creatine levels may represent a compensatory response to increased whole-body energy expenditure. Choline is a critical nutrient for central nervous system function, which is essential for the synthesis of the neurotransmitter acetylcholine and phosphatidylcholine (34). Notably, insufficient choline levels may indicate an increase in the body’s demand for this micronutrient and methyl donor. Choline plays a particularly pivotal role in hippocampal development and has been associated with pathways regulating gene methylation related to memory and cognitive functions (35). We speculate that choline deficiency leads to nervous system dysfunction in the acute stage and even adversely affects long-term nervous system development.

In addition to the aforementioned metabolites, S1P is a well-documented central component in immune cell function and vascular endothelium integrity (36). Circulating S1P plays critical physiological roles in limiting endothelial inflammation and regulating immune cell migration (13, 37). We observed elevated levels of serum S1P in severely infected mice and severe patients. The increase in circulating S1P may explain the endothelial barrier dysfunction and organ injuries seen in HFMD (38, 39). Additionally, acute tissue injury, vascular damage, and activation of glial cells in the brain are associated with upregulated S1P levels (40). Targeting directly or indirectly S1P signaling may be a useful novel strategy to treat EV-A71-associated encephalitis. Subsequently, we further explored the function of the S1P signaling pathway *in vivo*. MK571 improved the survival rates of infected mice, likely by inhibiting both constitutive and antigen-stimulated S1P release (41). Particularly, it may be associated with the S1P release of mast cells, as the number of degranulated mast cells was significantly increased in the brains, lungs, and skeletal muscles of the EV-A71-infected mice (42). Reducing the infiltration of WBC in brain tissue early may be the key to reducing nerve damage, which was related to the function of S1P in regulating immune cell migration and infiltration (13). We also investigated S1PR1, a receptor of S1P, using a selective agonist SEW2871. However, SEW2871 failed to improve the survival rates of infected mice. The absence of S1P agonist protective effect on infected mice may be attributed to the distinct and context-dependent functions of S1P binding to its various receptors. Five S1P receptors (S1PR1–5) mediate cellular responses to extracellular S1P, thereby regulating a wide array of physiological processes and pathological conditions across multiple organ systems (43). Upon binding to S1P, S1PRs undergo internalization, and the subsequent activation by small molecule agonists or albumin-bound S1P leads to downregulation of S1PR expression on the cell surface. This downregulation diminishes the responsiveness of target cells to further stimulation by circulating S1P (44). Consequently, we proposed that targeting the broader S1P metabolic pathway, rather than focusing solely on S1PR1 signaling, may offer more effective therapeutic strategies in treating EV-A71-associated encephalitis.

Although this study provides preliminary evidence supporting the therapeutic potential of modulating S1P metabolism in severe HFMD, there are several limitations that warrant further investigation. Specifically, the molecular mechanisms by which MK571 inhibits inflammatory cytokine production and leukocyte infiltration remain to be fully elucidated. Additionally, given the functional diversity of S1P receptors and their differential roles upon activation, further studies are essential to dissect the downstream signaling pathways involved in S1P-mediated regulation of inflammation and immune responses.

### Conclusions

We confirmed the potential of identifying HFMD patients who may eventually become severe cases based on the analysis of a panel of sera metabolites. Additionally, regulating S1P metabolism might provide an exploitable therapeutic target in EV-A71-associated HFMD.

## MATERIALS AND METHODS

### Study subjects

This study included children with EV-A71 infection who were hospitalized at the First Affiliated Hospital of Xinxiang Medical University from April to September 2017. The cases were diagnosed and defined based on the *Guidelines for the Diagnosis and Treatment of Hand, Foot and Mouth Disease* (2010 dition) from the China Ministry of Health (45). The patients included in this study were all laboratory-confirmed cases, but not clinically diagnosed cases relying solely on clinical observation. We confirmed EV-A71 infection in HFMD patients through successful detection of EV-A71 nucleic acid by qPCR, or at least four times increase of the titer of neutralizing antibody (IgM) against EV-A71 in the recovery phase and acute stage. To avoid confounding factors that could affect host metabolism, we excluded patients with other infections such as pneumonia, bronchitis, tuberculosis, influenza, sepsis, other viral diseases, congenital heart disease, nephrotic syndrome, immune system diseases, or a long history of glucocorticoid use. All HCs who were confirmed to exhibit normal clinical indicators and tested negative for EV-A71 through both qRT-PCR and serological IgM antibody assays were randomly selected from the asymptomatic healthy participants recruited during the health examination at Zhengzhou Central Hospital Affiliated to Zhengzhou University.

### Samples, clinical observation, and laboratory tests

Plasma samples from recruited patients and controls were collected using EDTA and sodium citrate anticoagulants. After centrifugation at 3,000 rpm for 10 min, the supernatant was aspirated and aliquoted into 500 µL centrifuge tubes and then immediately stored at −80°C for later use. Metabolomics analysis was performed on samples from 45 HCs, 45 patients with mild EV-A71 infection, and 39 patients with severe EV-A71 infection. Detailed information of the experimental methods and materials is available on the Supplementary file S1. Demographic, clinical, and laboratory data were collected through a review of medical records of the patients. Routine blood and biochemical tests were performed in the morning on the second day after hospitalization. The number of days since onset was determined based on parents’ reports or clinicians’ inquiries at the time of admission.

### Animal experiment *in vivo*

The animal experiments involving EV-A71 infection were conducted following the procedures described in a previous study (46, 47). Five-day-old ICR mice were inoculated intraperitoneally (i.p.) with a lethal dose of EV-A71 stains (10^7^ TCID50 ZZ1350, accession number: OP806304). Six hours post infection, the mice received an i.p. injection of MK571 (dissolved in water) at proper doses (1, 5, 10 mg/kg) (*N* = 10–17 per group) (48). The control group was administered an equivalent amount of water. Following treatment, the mice were monitored daily for clinical symptoms and survival rates until 15 days post-infection (dpi). MK571, an inhibitor of S1P, was purchased from Med Chem Express LLC (Cat#: HY-19989). In the experiment to regulate S1PR1 signaling pathways, SEW2871 (Cat#: HY-W008947), a highly selective S1PR1 agonist, was administered to the infected mice.

### Mouse blood analysis

For the measurement of serum differential metabolite levels in infected mice, serum samples were collected at 2 and 5 dpi. The concentrations of serum S1P (Shanghai Enzyme-linked Biotechnology Co., Ltd., Cat#: ml062988), L-phenylalanine (Elabscience Biotechnology Co., Ltd., Cat#: E-BC-K846-M), choline (Shanghai COIBO Biotechnology Co., Ltd., Cat#: CB10904-Mu), and creatine (Beijing Solarbio Science & Technology Co., Ltd. Cat#: BC4920) were measured using corresponding kits according to the manufacturer’s instructions. Blood samples were collected from four groups of mice (control, MK571, EV-A71, and EV-A71 + MK571) after they were sacrificed by inhaling excessive isoflurane at 2 dpi. The routine blood was analyzed using an automatic blood cell analyzer (Shenzhen Mindray Biomedical Electronics Co., Ltd.., BC-2800 Vet).

### Histopathological analysis

At 2 and 5 dpi, three mice from each group were randomly selected to be euthanized. Brain samples were collected and fixed in 4% paraformaldehyde for 48 h. Following fixation, the tissues were embedded in paraffin, sectioned into 5 µm slices, and subsequently stained with hematoxylin and eosin (H&E) for histopathological analysis.

### Flow cytometry analysis

At 2 dpi, the mouse brains were harvested aseptically, and a single-cell suspension was prepared in PBS. The single cells were blocked with an anti-mouse CD16/32 antibody for 15 min at 4°C, followed by incubation with fluorescently labeled PerCP anti-mouse CD45 antibody (BioLegend, Cat#: 157208) and FITC-LIVE/DEAD (Thermo Fisher Scientific Co., Ltd, Cat#: L34969) at 4°C for 50 min in the dark. After washing twice with PBS and resuspending, the numbers of CD45 positive cells were detected using a flow cytometer (Agilent, Novocyte3008R).

### Quantitative real-time PCR

To determine the transcriptional levels of certain cytokines, total RNA was extracted from brain tissues using TRIzol reagent (Invitrogen Corporation, California, USA). One microgram of total RNA was reverse transcribed into cDNA with Hifair II 1st Strand cDNA Synthesis Kit (Yeasen, Co., Ltd, Shanghai, China). Quantitative real-time PCR (qPCR) was performed with Hieff qPCR SYBR Green Master Mix (Yeasen, Co., Ltd, Shanghai, China) by a fluorescent quantitation PCR instrument (Bio-Rad CFX Opus 96 Real-Time PCR System). The primer sequences used in this study were shown as follows: interleukin-6 (IL-6), forward: CTGCAAGAGACTTCCATCCAG, Rrverse: AGTGGTATAGACAGGTCTGTTGG; IL-1β, forward: GAAATGCCACCTTTTGACAGTG, reverse: TGGATGCTCTCATCAGGACAG; tumor necrosis factor α (TNFα), forward: CCTGTAGCCCACGTCGTAG, reverse: GGGAGTAGACAAGGTACAACCC; monocyte chemoattractant protein-1 (MCP-1), forward: TAAAAACCTGGATCGGAACCAAA, reverse: GCATTAGCTTCAGATTTACGGGT; mouse β-actin, forward: GTGCTATGTTGCTCTAGACTTCG, reverse: ATGCCACAGGATTCCATACC. Expression levels of mRNAs were quantified as relative values compared to β-actin using the 2^−ΔΔ*Ct*^ method. The viral loads were calculated as described in our previous study (49).

### Statistical analysis

All the analyses were performed using R software version 4.1.1 or GraphPad Prism version 8.3 software (San Diego, CA, USA). Chi-square test was used to evaluate the differences of categorical data, while continuous variables were compared by one-way analysis of variance or Mann-Whitney *U* test according to data distribution. Multiple comparisons among three groups were performed using two-sample *t* tests with Bonferroni’s adjustment or Kruskal-Wallis test. Pearson correlation analysis was performed to analyze the correlation between two different indicators. Categorical variables were expressed as percentages, while continuous variables were presented as mean ± standard deviation or median with interquartile range. The survival rates of each group were analyzed by the Mantel-Cox log rank test. The threshold for significance was *P* < 0.05.

## Data Availability

The data sets supporting the conclusions of this article are included within the article and its supplemental files. Additionally, the metabolism data set is available in the MetaboLights database under identifier MTBLS11656.

## SUPPLEMENTAL MATERIAL

Figure S1 aac.01272-24-s0001.tif Differential metabolic profiles and metabolic pathways across different comparison strategies.

Figure S2 aac.01272-24-s0002.tif Bayesian model, random forest model, and artificial neural networks analysis were constructed for calculating mild group and severe group diagnosis score.

Figure S3 aac.01272-24-s0003.tif Correlation analysis between differentiated metabolites and several clinical indicators with statistical difference.

Figure S4 aac.01272-24-s0004.tif Serum differential metabolite levels and the effect evaluation of MK571.

Figure S5 aac.01272-24-s0005.tif Proinflammatory and pathological change of mouse brains at 5dpi.

Figure S6 aac.01272-24-s0006.tif S1PR1 agonist, SEW2871, could not decrease the fatality rate of infected mice.

Supplemental material aac.01272-24-s0007.doc Supplemental methods, Table S1, and legends for Fig. S1 to S6.

ASM does not own the copyrights to Supplemental Material that may be linked to, or accessed through, an article. The authors have granted ASM a non-exclusive, world-wide license to publish the Supplemental Material files. Please contact the corresponding author directly for reuse.
