# Supplementary figures and images for: Metabolomic analysis reveals an important role of sphingosine 1-phosphate in the development of HFMD due to EV-A71 infection
Source: Antimicrob Agents Chemother. 2024 Dec 18;69(2):e01272-24. doi: 10.1128/aac.01272-24 (PMC11823611; doi:10.1128/aac.01272-24)

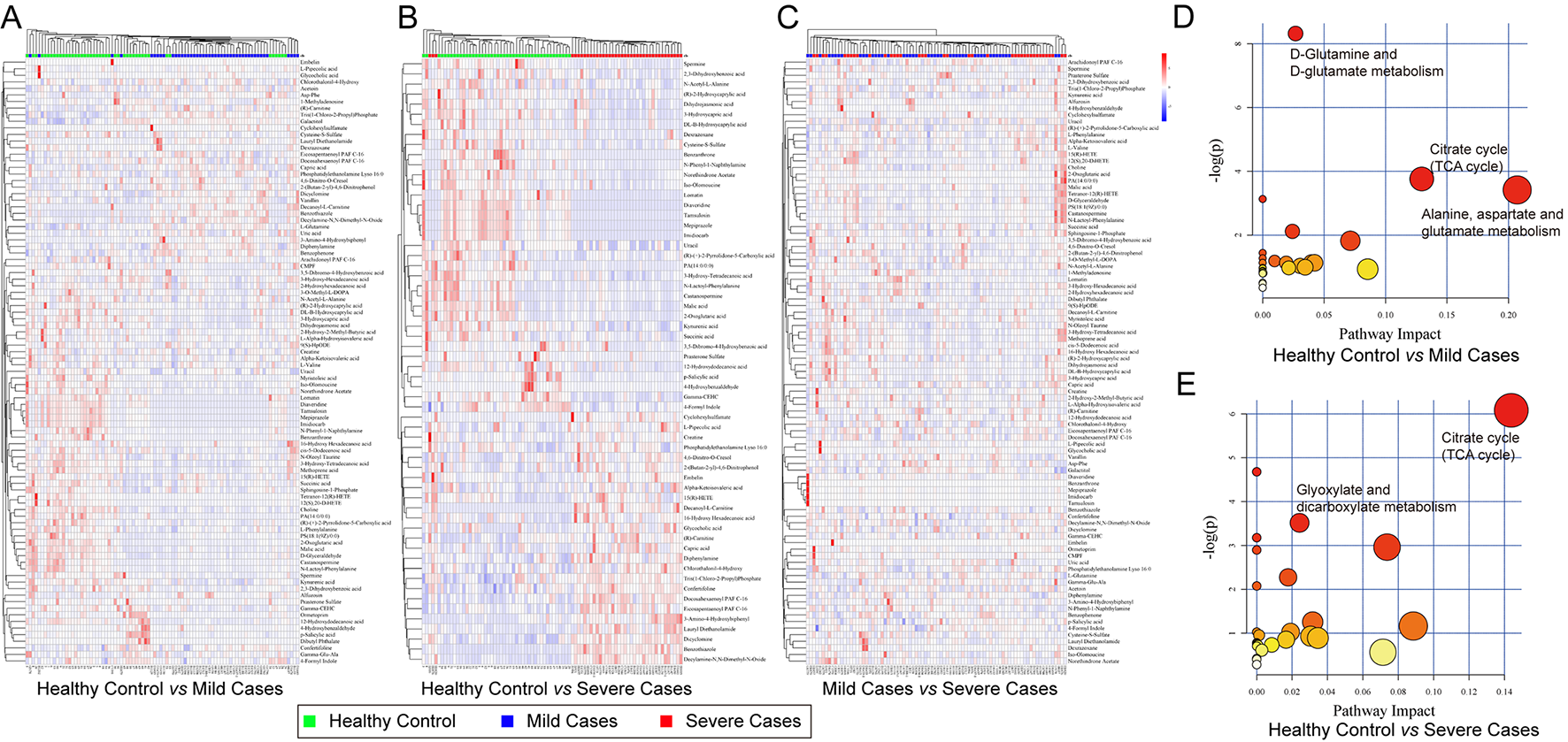

Supplement: Figure S1 — Differential metabolic profiles and metabolic pathways across different comparison strategies. [file aac.01272-24-s0001.tif]

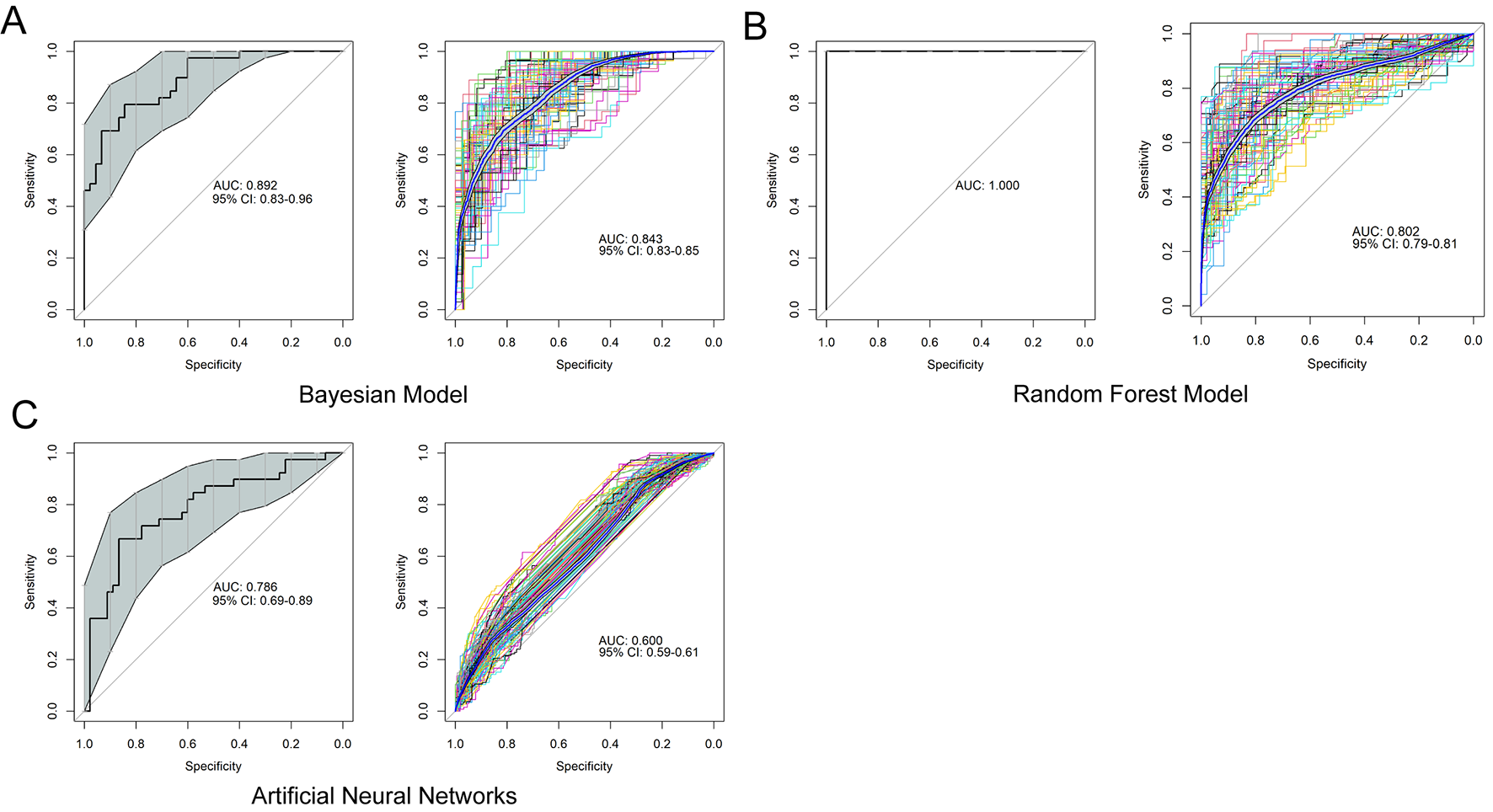

Supplement: Figure S2 — Bayesian model, random forest model, and artificial neural networks analysis were constructed for calculating mild group and severe group diagnosis score. [file aac.01272-24-s0002.tif]

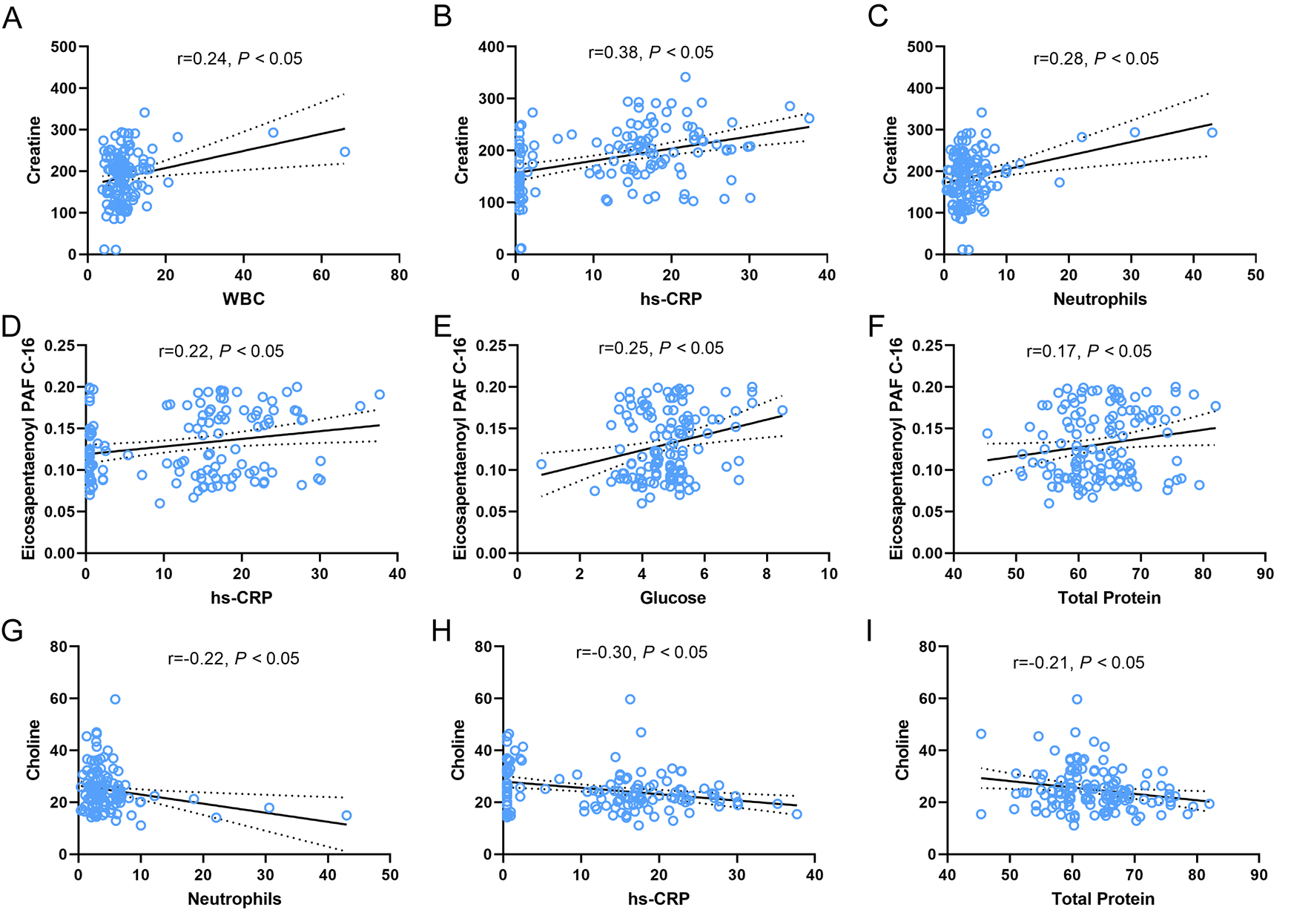

Supplement: Figure S3 — Correlation analysis between differentiated metabolites and several clinical indicators with statistical difference. [file aac.01272-24-s0003.tif]

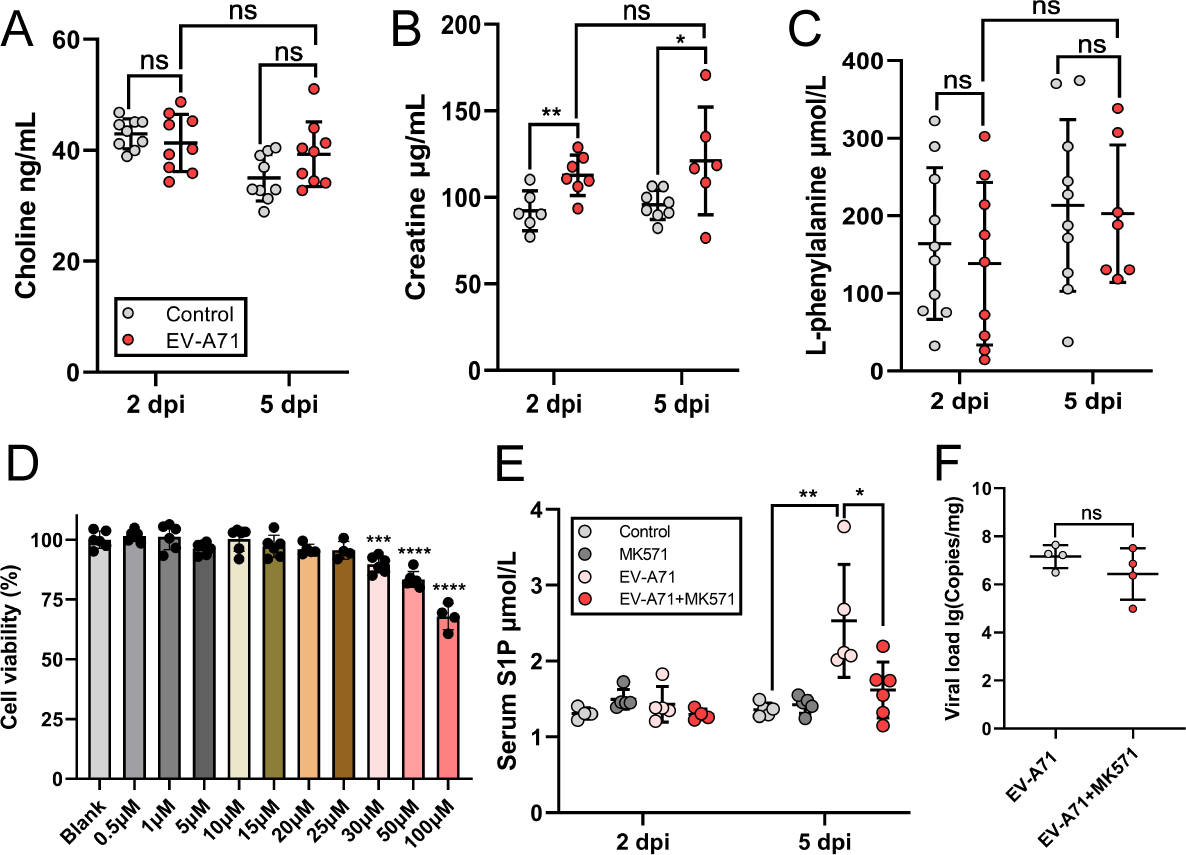

Supplement: Figure S4 — Serum differential metabolite levels and the effect evaluation of MK571. [file aac.01272-24-s0004.tif]

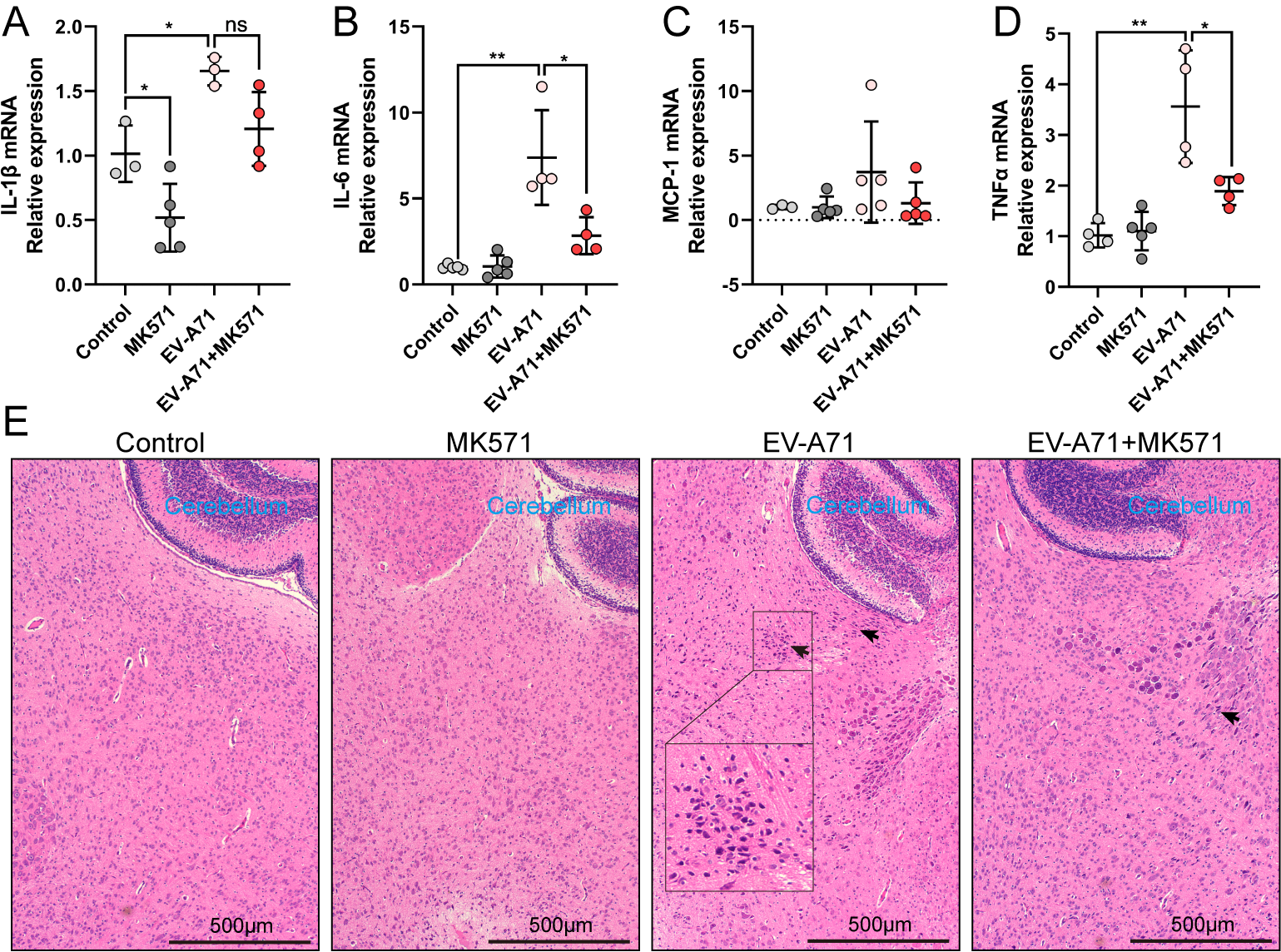

Supplement: Figure S5 — Proinflammatory and pathological change of mouse brains at 5dpi. [file aac.01272-24-s0005.tif]

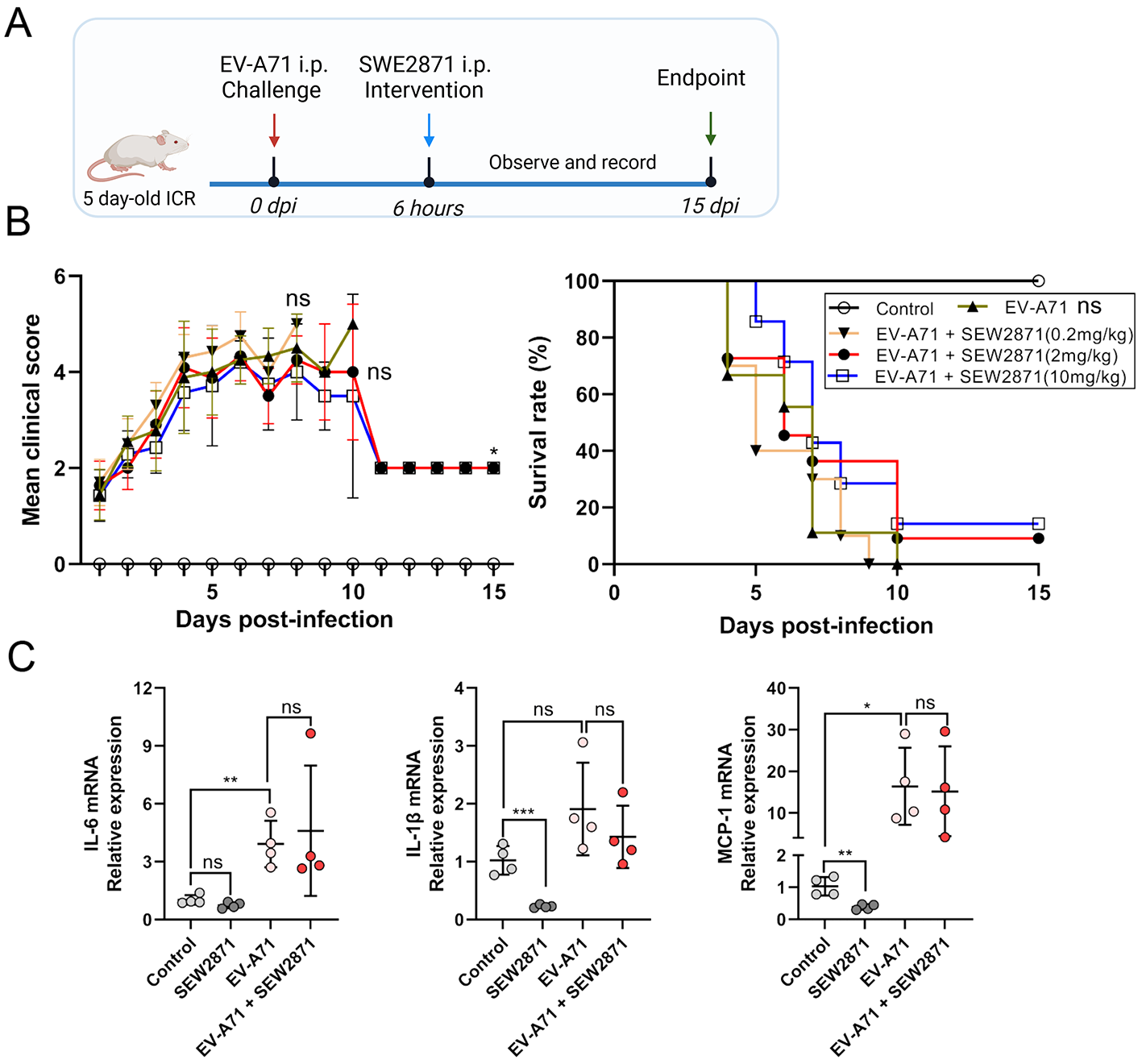

Supplement: Figure S6 — S1PR1 agonist, SEW2871, could not decrease the fatality rate of infected mice. [file aac.01272-24-s0006.tif]
