# Supplementary material for: Metabolomic analysis reveals an important role of sphingosine 1-phosphate in the development of HFMD due to EV-A71 infection
Source: Antimicrob Agents Chemother. 2024 Dec 18;69(2):e01272-24. doi: 10.1128/aac.01272-24 (PMC11823611; doi:10.1128/aac.01272-24)
Supplement: Supplemental material — Supplemental methods, Table S1, and legends for Fig. S1 to S6. [file aac.01272-24-s0007.doc]

**Metabolomic Analysis Reveals an Important Role of Sphingosine 1-phosphate in the Development of HFMD due to EV-A71 Infection**

Wangquan Ji^a^, Dejian Dang^b^, Guangyuan Zhou^c^, Ling Tao^c^, Tiantian Sun^a^, Dong Li^a^, Cheng Cheng^a^, Huifen Feng^b^, Jinzhao Long^a^, Shuaiyin Chen^a^, Haiyan Yang^a^, Guangcai Duan^a^, Yuefei Jin^a, d^*

^a^ Department of Epidemiology, College of Public Health, Zhengzhou University, Zhengzhou 450001, Henan, China;

^b^ Department of Infection Control, The Fifth Affiliated Hospital of Zhengzhou University, Zhengzhou 450052, Henan, China;

^c^ School of Public Health, Xinxiang Medical University, Xinxiang 453003, Henan, China.

^d^ Pingyuan Laboratory, Xinxiang 453007, China.

*Correspondence should be addressed to Yuefei Jin ([jyf201907@zzu.edu.cn](mailto:jyf201907@zzu.edu.cn)).

**Running title:** Metabolomic Characterization of HFMD

**Supplemental Methods and Materials**

***Metabolite extraction from plasma samples***

The experimental steps are as follows: (1) Thaw all samples at 4℃; (2) Transfer 200 µL of each sample into 1.5 ml centrifuge tubes; (3) Add 800 µL of methanol (pre-cooled at -20℃) to each tube and vortex for 60 s; (4) Centrifuge for 10 min at 12 000 rpm 4℃ and transfer all supernatant in each tube into another 1.5 mL centrifuge tube, samples were blow-dried by vacuum concentration; (5) Dissolve samples with 300 μL methanol aqueous solution (4:1, 4℃), Filter through a 0.22 µm membrane and obtain the prepared sample extracts for LC-MS; (6) For the quality control (QC) samples, take 20 µL from each prepare sample extract and mix (These QC samples were used to monitor deviations of the analytical results from these pool mixtures and compare them to the errors caused by the analytical instrument itself); (7) Use the rest of the samples for LC-MS test.

***Metabolite measurement***

Processed plasma samples were analyzed by ultrahigh performance liquid chromatography-mass spectrometry (UPLC-MS). Chromatographic separation was accomplished in a Thermo Ultimate 3000 system equipped with an ACQUITY UPLC® HSS T3 (150×2.1 mm, 1.8 µm, Waters) column maintained at 40℃. Gradient elution of analytes was carried out with 0.1% formic acid in water (A) and 0.1% formic acid in acetonitrile (B) or 5mM ammonium formate in water (C) and acetonitrile (D) at a flow rate of 0.25mL/min. Injection of 2 μL of each sample was done after equilibration. An increasing linear gradient of solvent B (v/v) was used as follows: 0~1 min, 2% B/D; 1~9 min, 2%~50% B/D; 9~12 min, 50%~98% B/D; 12~13.5 min, 98% B/D; 13.5~14 min, 98%~2% B/D; 14~17 min, 2%B/D. The ESI-MSn experiments were executed on the Thermo Q Exactive Focus mass spectrometer with the spray voltage of 3.8 kV and -2.5 kV in positive and negative modes, respectively. Sheath gas and auxiliary gas were set at 45 and 15 arbitrary units, respectively. The capillary temperature was 325 ℃. The Orbitrap analyzer scanned over a mass range of m/z 81-1 000 for full scan at a mass resolution of 70 000. Data dependent acquisition (DDA) MS/MS experiments were performed with HCD scan. The normalized collision energy was 30 eV. Dynamic exclusion was implemented to remove some unnecessary information in MS/MS spectra. During acquisition, one QC sample was run after every 10 samples.

***Metabolomics profiling and data processing***

The UPLC-MS raw data were converted to mzXML file using the ProteoWizard software (v3.0.8789, Pala Alto, California, USA), and then were processed for peak detection, extraction, alignment, and integration by XCMS package (La Jolla, California, USA). The peaks were aligned and normalized to the sum of all the detected peaks to minimize the impact of both noise and high variance of the variables. After these transformations, principal component analysis (PCA) was performed to visualize the distribution and the grouping of the samples. Next, to visualize group separation, partial least squares discriminant analysis (PLS-DA) was carried out. To find differential metabolites between group comparisons, the orthogonal projections to latent structure-discriminant analysis (OPLS-DA) model was introduced to determine the maximum separation between different kinds of samples according to the sample classification information. The metabolites with variable importance in projection (VIP) > 1 was included for further analysis. The Benjamini-Hochberg procedure was applied to control the false discovery rate due to multiple testing. p < 0.05 was considered statistically significant, and the differential metabolites screened by multidimensional statistical analysis were verified. Variables with VIP > 1 for multi-dimensional statistics and p < 0.05 for single-dimensional statistics and fold change ≥ 1.5 or ≤ 0.667 between two groups were considered as significantly altered metabolites (or differential). The names of the metabolites were identified by searching the online human metabolome database via aligning the molecular mass data (m/z). The dataset was scaled by the heatmap package in R (v3.3.2) to obtain a hierarchical cluster map of the relative quantitative values of metabolites. In order to characterize the correlation of differential metabolites, Pearson correlation analysis was performed on the peak intensities corresponding to differential metabolites in this study. MetaboAnalyst platform combined with the online Kyoto Encyclopedia of Genes and Genomes (KEGG) classifications were used for pathway analysis of differential metabolites. The differential metabolites were mapped into their biochemical pathway. Pathway significance was based on the total number of metabolites that map to a pathway and their respective significances; a pathway was considered perturbed if the number of significant metabolites (i.e., hits) was ≥ 2 and the impact value was ≥ 0.10 and raw p < 0.10. Raw p values were determined on the basis of the number of hits and total number of compounds in the pathway. An impact value ≥ 0.10 indicates that the altered pathway has a clear impact.

A backward stepwise regression based on Akaike information criterion was used to get the final model. Different to the regression model, the variables in random forest model (random Forest 4.7-1.1 package) were screened by a fivefold cross-validation. The cross-validation error curves (average of five test sets each) were acquired from five trials of the fivefold cross-validation, and the minimum error in the averaged curve plus the standard deviation at that point was used as the cutoff. The sets (≤3) of variables with the error less than the cut-off value were listed and the set with the smallest number of variables as the optimal set. ROC curve analysis (pROC 1.17.0.1 package) was used to assess the diagnostic performance (sensitivity and specificity) of these identified panels. The area under the ROC curve (AUC) was calculated to estimate the degree of discrimination between severe and mild cases.

***Quantitative determination of differential metabolites***

In this study, an ultra-high-performance liquid chromatography coupled with triple quadrupole electrospray tandem mass spectrometry (UHPLC-TQ-MS/MS) (Waters Corporation, ACQUITY-I UPLC/Xevo TQS, USA) method was developed and validated for the simultaneous determination of six differential metabolites. The raw data generated by UHPLC-TQ-MS/MS were processed for peak detection, extraction, integration and quantification for each metabolite using MassLynx software from Waters (v4.1, Waters, Milford, MA, USA). Standard products Eicosapentaenoyl PAF C-16 (Cat#ZC-23012), Alpha-Ketoisovaleric acid (Cat#198994), L-Phenylalanine (Cat#P1150000), Sphingosine 1-phosphate (Cat#73914), Creatine (Cat#1150320), and Choline (Cat#PHR1251) were purchased from Shanghai Zhenzhun Biotechnology Co., Ltd.

**Table S1 The basic characteristics and laboratory indicators of EV-**A71 patients

| Variables | Healthy Control (n=45) | Mild (n=45) | Severe (n=39) | *χ2/F* | *P* |
| --- | --- | --- | --- | --- | --- |
| Gender (male) n, % | 28(62.2%) | 31(68.9%) | 23(59%) |  | 0.344 |
| Age (month) | 28(19, 40.25) | 25(15.25, 38) | 23(14, 33.75) |  | 0.232 |
| WBC (10^9^/L) | 7.21(6.4, 8.49) | 9.2(8.1, 10.2)*** | 10.4(7.65, 14.1)*** | 25.694 | <0.001 |
| RBC (10^12^/L) | 4.19(3.92, 4.45) | 4.09(3.9, 4.39) | 4.26(3.915, 4.41) | 0.855 | 0.652 |
| Platelets (10^9^/L) | 322.4±69.66 | 332.58±106.79 | 349.21±86.55 | 0.955 | 0.387 |
| HGB (g/L) | 107(99,116) | 109(101,118) | 113(106.5,119.5)* | 6.458 | 0.040 |
| HCT (%) | 32.5±4.03 | 31.51±3.63 | 32.69±3.43 | 1.260 | 0.287 |
| MCV | 80.7(74.8,84.2) | 78(74.7,801) | 77.6(73.85,81.2) | 4.958 | 0.084 |
| MCH (pg) | 26.2(24.1,27.6) | 27.2(24.6,28.9) | 27.8(26.4,28.35)* | 6.616 | 0.037 |
| MHC (g/L) | 327(319,335) | 353(328,365)*** | 349(330.5,360.5)*** | 25.265 | <0.001 |
| Neutrophil ratio (%) | 41.8(30.9,48.2) | 36(28,56) | 52(34,62.5) | 5.584 | 0.061 |
| Lymphocyte ratio (%) | 50.1(42.5,60) | 54(37,67) | 43(31.5,57.5) | 5.934 | 0.051 |
| Monocyte ratio (%) | 5.8(5.3,7.6) | 6(4,8) | 5(3.5,7.5) | 1.257 | 0.533 |
| Eosinophil ratio (%) | 1.6(0.8,3.6) | 1(1,2) | 1(0,1)***## | 19.510 | <0.001 |
| Basophil ratio (%) | 0.4(0.1,0.4) | 1(0.8,1.4)*** | 0.8(0,1.2)* | 18.481 | <0.001 |
| Neutrophil count (10^9^/L) | 2.93(2.09,3.93) | 3.1(2.4,4.6) | 6(2.85,6.85)***# | 15.106 | <0.001 |
| Lymphocyte count (10^9^/L) | 3.28(3.07,4.69) | 4.6(2.8,5.8) | 4.1(3.05,5.65) | 4.911 | 0.086 |
| Monocyte count (10^9^/L) | 0.47(0.39,0.58) | 0.52(0.3,0.75) | 0.5(0.4,0.895) | 2.498 | 0.287 |
| Eosinophil count (10^9^/L) | 0.13(0.05,0.28) | 0.1(0.06,0.19) | 0.08(0.015,0.1)**# | 10.196 | 0.006 |
| Basophil count (10^9^/L) | 0.03(0.01,0.04) | 0.08(0.03,0.11)*** | 0.06(0,0.1)* | 16.772 | <0.001 |
| MPV | 8.3(7.7,8.9) | 7.5(7.2,7.9)*** | 7.5(7.2,7.9)*** | 19.407 | <0.001 |
| Hs-CRP (mg/L) | 0.5(0.5,0.89) | 1.6(0.7,8.4)** | 2.22(0.495,5.005)* | 10.751 | 0.005 |
| K (mmol/L) | 4.4(4.2,4.6) | 4.6(4.38,4.83) | 4.57(4.28,5.035) | 4.405 | 0.111 |
| Na (mmol/L) | 138(137,139) | 137(135,138)* | 135(134,138.5)** | 13.139 | 0.001 |
| Cl (mmol/L) | 106(104,107) | 104.3(101,106.1)* | 102.9(99.85,105.5)*** | 14.832 | <0.001 |
| Ca (mmol/L) | 2.44(2.35,2.59) | 2.39(2.35,2.44) | 2.39(2.345,2.49) | 3.240 | 0.198 |
| Glucose (mmol/L) | 5(4.9,5.3) | 4(3.6,4.53)*** | 5(4.16,6.125)### | 26.944 | <0.001 |
| Urea (mmol/L) | 3.88±0.88 | 3.61±0.96 | 3.67±1.26 | 0.872 | 0.421 |
| Creatinine (μmol/L) | 23(21.1,26.5) | 21(17.4,24.8) | 22.6(19.15,29.1) | 3.035 | 0.219 |
| Uric acid (μmol/L) | 241.2(218.4,271.5) | 252(209,297) | 230(188.5,301.5) | 1.054 | 0.591 |
| Total protein (g/L) | 61.6(59.7,65.4) | 60.7(57.8,65.7) | 67(62.75,71.85)**### | 18.014 | <0.001 |
| Albumin (g/L) | 42.9(41.2,46) | 45.4(42.8,47.6) | 45.6(44.55,48.5)** | 11.742 | 0.003 |
| Globulin (g/L) | 18.4(16.9,20.6) | 15.7(13.8,17.8)** | 21.3(17.3,24)### | 23.831 | <0.001 |
| TBIL (μmol/L) | 8(5.3,10.2) | 6(4.4,7.5) | 6(4.1,8.2)* | 7.190 | 0.027 |
| DBIL (μmol/L) | 1.8(1.3,2.9) | 2.6(1.9,3.4)** | 2.5(2.2,3.35)* | 11.866 | 0.003 |
| IBIL (μmol/L) | 5.6(3.4,7.8) | 3.4(2.5,4.5)*** | 3(1.8,5.05)*** | 22.541 | <0.001 |
| ALT (U/L) | 18(15,28) | 14(12,20)* | 14(10,20.5)** | 11.487 | 0.003 |
| AST (U/L) | 32(28,37) | 34(29,44) | 32(28,39) | 2.347 | 0.309 |
| GGT (U/L) | 12(9,14.5) | 12(10,18) | 14(11,23) | 3.486 | 0.175 |
| ALP (U/L) | 232(199.4,263) | 255(229,303)* | 271(215.5,325)* | 11.142 | 0.004 |
| LDH (U/L) | 259(242,293) | 269(230,294) | 254(236,276.5) | 1.593 | 0.451 |
| αHBDH (U/L) | 239(220,256) | 190(167,225)*** | 191(178,221)*** | 24.975 | <0.001 |
| CK (U/L) | 116(91,143) | 79(63,101)*** | 76(53.5,93.5)*** | 22.125 | <0.001 |
| CK-MB (U/L) | 25(22,34) | 23(20,27) | 22(19,25.5)* | 8.130 | 0.0171 |

Categorical variables were expressed as percentages, while clinical characteristics and laboratory findings were presented as mean ± standard deviation or median with interquartile range. *, **, *** indicate healthy control vs mild or severe *P*<0.05, *P*<0.01 and *P*<0.001; #, ##, ### indicate mild vs severe *P*<0.05, *P*<0.01 and *P*<0.001.

WBC: white blood cells; RBC: red blood cells; HGB: hemoglobin; HCT: hematocrit; MCV: mean red blood cell volume; MCV: mean hemoglobin; MCH: mean content of hemoglobin; Hs-CRP: high-sensitivity C-reactive protein; TBIL: total bilirubin; DBIL: direct bilirubin; IBIL: indirect bilirubin; ALT: alanine aminotransferase; AST: aspartate aminotransferase; GGT: glutamyltransferase; ALP: alkaline phosphatase; LDH: lactate dehydrogenase; αHBDH: alpha-hydroxybutyric acid; CK: creatine kinase; CK-MB: creatine kinase isoenzymes.

**Figure Legends of all Supplemental Material Files**

**Supplemental file 1.** Supplemental methods and materials, basic characteristics and laboratory indicators of EV-A71 patients, and figure legends of all supplemental material files.

**Figure S1. Differential metabolic profiles and metabolic pathways across different comparison strategies.** (**A-C**) Heatmap visualization of hierarchical clustering based on significantly altered metabolites. (**D-E**) Metabolic pathway analysis was launched using identified differential metabolites.

**Figure S2.** (**A**) Bayesian model, (**B**) Random forest model, and (**C**) Artificial neural networks analysis were constructed for calculating mild group and severe group diagnosis score.

**Figure S3**. Correlation analysis between differentiated metabolites (**A-C**, Creatine; **D-F**, Eicosapentaenoyl PAF C-16; **G-I**, Choline) and several clinical indicators with statistical difference.

**Figure S4. Serum differential metabolite levels and the effect evaluation of MK571.** Serum samples were collected at 2 dpi and 5 dpi. The concentrations of serum (**A**) choline, (**B**) creatine and (**C**) L-phenylalanine were measured using corresponding kits according to the manufacturer’s instructions (N=6-10 per group). (**D**) Cell viability after MK571 treatment was assessed by CCK8 assay (Shanghai huaxiang biological Technology Co., LTD, Cat#: B2201-500T) based on RD cell. (**E**) Serum S1P levels of mice from four groups were measured by ELISA at 2dpi and 5dpi (N=4-6 per group). (**F**) The viral loads of brains were measured between the EV-A71 infection group and the MK571 intervention group at 5 dpi. *, *P* < 0.05; **, *P* < 0.01; ***, *P* < 0.001; **** *P* <0.0001; ns, no statistical differences.

**Figure S5. Proinflammatory and pathological change of mouse brains at 5dpi.** qPCR was conducted to measure the gene expression levels of some typical inflammatory cytokines (**A**, IL-1β; **B**, IL-6; **C**, MCP-1; **D**, TNFα) in the brains EV-A71-infected and mock mice with MK571 interventions at 5 dpi (N=3-4 per group). (**E**) Pathological changes in the brains of mice at 5 dpi were evaluated by H&E staining. The black arrows indicate characteristic pathological changes, including gliacyte proliferation and neuronal degeneration. The scales are indicated in these images. *, *P* < 0.05; **, *P* < 0.01; ns, no statistical differences.

**Figure S6. S1PR1 agonist, SEW2871, couldn’t decrease the fatality rate of infected mice.** (**A**). A schematic diagram above the panel was added to explain the animal experiment. (**B**). Mean clinical score and survival rate were monitored daily for 15 days (N=7-11 per group, *vs* EV-A71). (**C**) The transcriptional levels of IL-6, Il-1β and MCP-1 in brain tissue were measured by qPCR (N=4 per group). *, *P* < 0.05; **, *P* < 0.01; ***, *P* < 0.001; ns, no statistical differences. Created with BioRender.com.
